# Community Pharmacists’ Views and Practices Regarding Natural Health Products Sold in Community Pharmacies

**DOI:** 10.1371/journal.pone.0163450

**Published:** 2016-09-23

**Authors:** Ubaka Ogbogu, Candace Necyk

**Affiliations:** 1 Faculty of Law, University of Alberta, Edmonton, Alberta, Canada; 2 Faculty of Pharmacy and Pharmaceutical Sciences, University of Alberta, Edmonton, Alberta, Canada; Royal College of Surgeons in Ireland, IRELAND

## Abstract

**Background:**

Reports of regulatory and evidentiary gaps have raised concerns about the marketing and use of natural health products (NHPs). The majority of NHPs offered for sale are purchased at a community pharmacy and pharmacists are “front-line” health professionals involved in the marketing and provision of NHPs. To date, the involvement of pharmacists in pharmacy care involving NHPs and the degree to which concerns over the safety, efficacy, marketing and regulation of NHPs are addressed in pharmacy care in Canada have not been studied.

**Methods:**

Using Qualtrics, a web-based data collection and analysis software, and a study instrument made up of fifteen (15) open-ended, closed and rating scale questions, we surveyed the attitudes and practices of 403 community pharmacists in the Canadian province of Alberta regarding NHPs offered for sale in community pharmacies.

**Results:**

The majority of pharmacists surveyed (276; 68%) recommend NHPs to clients sometimes to very often. Vitamin D, calcium, multivitamins, prenatal vitamins, probiotics and fish oil and omega-3 fatty acids were the most frequently recommended NHPs. The most common indications for which NHPs are recommended include bone and musculoskeletal disorders, maintenance of general health, gastrointestinal disorders and pregnancy. Review articles published in the Pharmacist’s Letter and Canadian Pharmacists Journal were the primary basis for recommending NHPs. The majority of pharmacists surveyed (339; 84%) recommend the use of NHPs concurrently with conventional drugs, while a significant number and proportion (125; 31%) recommend alternative use. Pharmacists in the study overwhelmingly reported providing counselling on NHPs to clients based on information obtained mainly from the Natural Medicines Comprehensive Database.

**Conclusions:**

The study findings indicate a high prevalence of pharmacy care relating to NHPs among study participants. Although pharmacists’ practices around NHPs are consistent with the existing licensing framework, we found some involvement in problematic practices that necessitate further research and potential policy scrutiny. The study also uncovered patterns of recommendations, including sources relied on in recommending NHPs and in providing counselling to patients, that raise concerns about the quality and credibility of NHP-related care provided to pharmacy patrons.

## Introduction

Natural health products (NHPs) are “naturally occurring substances,” including vitamins, minerals, herbal remedies, homeopathic medicines, traditional medicines, probiotics, amino acids and fatty acids that are “used and marketed for…the prevention or treatment of an illness or condition, the reduction of health risks, or the maintenance of good health” [1]. Studies published in the present decade have reported increased use of NHPs both within Canada and internationally [2–5]. A recent national survey in Canada found that 73% of adult Canadians took at least one NHP in 2010 [5]. A third of the individuals in the same survey reported taking three or more NHPs concurrently [5]. Uses of NHPs in the pediatric population have also been reported [6]. Reasons for using NHPs are many and varied, including an increased interest in natural approaches to disease prevention, the perception or belief that NHPs are better than conventional pharmaceutical drugs, media advertising, and recommendations from family and friends [5,7].

A common rationale for the prevalent use of NHPs is that consumers believe that they are either safe or safer than pharmaceuticals [5,7]. However, the safety profiles of commonly used NHPs are understudied and there is limited high quality, credible research evidence to support the safety of NHPs [8–11]. Adverse events have been documented for a number of NHPs, especially when used concurrently with conventional drugs [12–13]. Such adverse events include intrinsic effects related to pharmacologic action, toxicity, dosage and interaction with other drugs, as well as extrinsic effects related to contamination issues and manufacturing and labelling errors [14–16]. For example, ephedra, a natural health product marketed as a stimulant and weight-loss supplement, has been linked to hypertension, tachycardia and anxiety [17]. In 2004, the United States Food and Drug Administration (FDA) banned the sale of dietary supplements containing ephedra due to the “unreasonable risk of…cardiovascular complications and…death” [18]. Another well-known natural health product, St. John’s wort (*Hypericum perforatum*), is highly interactive and can lead to adverse effects when taken concurrently with other medications such as antidepressants and oral contraception [17]. Extrinsic effects have been reported for black cohosh (*Actaea racemosa*), where hepatotoxicity may be linked to Chinese *Actaea* species found in the product rather than black cohosh itself [19].

Evidence on the efficacy of NHPs is also limited and inconclusive. While some NHPs have been shown to be effective for treating specific indications, such as the use of calcium and Vitamin D for preventative treatment of osteoporosis in the elderly [20], others lack good quality evidence to support the uses for which they are marketed [21–22]. For example, glucosamine is marketed as a treatment for osteoarthritis. It is available by prescription in Europe and as an over-the-counter purchase in North America [21–22]. A 2008 Cochrane review of randomized controlled trials that evaluated the effectiveness of glucosamine for the treatment of osteoarthritis concluded that one specific type, glucosamine sulphate, administered orally at a specified dose, produced some benefit in pain management [22]. However, the review also found that “if only the best designed studies” are considered, this benefit “is no longer present” and that “the high quality studies [included in the review] showed that pain improved about the same whether people took glucosamine or fake pills” [22].

Given the popularity of, and demand for, NHPs, it is not surprising that they have entered the market despite limited evidence of safety or efficacy. There are currently over 70,000 NHPs licensed for sale in Canada and NHPs are the top over-the-counter category in terms of sales. Canadian consumers spend about $8.83 per capita on NHPs and the total yearly spending on popular NHPs including vitamins, dietary supplements and herbal remedies exceeded $1.5 billion in 2015 [23–26]. In addition, most major pharmacy chains in Canada now have aisles and counters dedicated to NHPs.

The increased availability of NHPs is partly aided by a regulatory system that imposes less scrutiny on NHPs prior to market entry compared to that imposed upon pharmaceuticals. NHPs offered for sale in Canada must be licensed and approved by the Natural and Non-Prescription Health Product Directorate (NNHPD), a branch of Health Canada. However, evidentiary and licensing requirements for NHPs are significantly more relaxed relative to the requirements for the licensing of pharmaceuticals [25–28]. While this approach may seem justified, given that NHPs are sold over-the-counter and without a prescription, it also allows for faster, easier and unencumbered market entry for NHPs. The licensing system has been described as “a joke” [29] and criticized for creating “a loophole through which manufacturers can sell a product with implied health benefits without having to obtain the supporting scientific evidence that would be needed if it were sold as a drug” [28]. A recent investigation conducted by CBC *Marketplace* into the licensing of NHPs in Canada highlights the latter criticism [29]. The investigation revealed that Health Canada approved a fake homeopathic product designed to provide “effective relief of fever, pain and inflammation” in children based solely on a claim of effectiveness derived from a 1902 homeopathic reference book [29]. The controversy and widespread public outcry that followed the *Marketplace* investigation led to changes to Health Canada’s policies around labeling and health claims for nosodes and homeopathic cough, cold and flu products for children [30].

NHPs approved for sale in Canada are assigned a Natural Health Product Number (NPN) (or Homeopathic Medicine Number (DIN-HM) in the case of homeopathic medicines) that must appear on the packaging of the product. It is presently unclear how product approval numbers and the licensing process for NHPs are perceived by members of the public and health professionals involved in the marketing of NHPs. However, concerns have been raised that the licensing process and issuance of product numbers may convey the impression that licensed NHPs are safe and efficacious for the purposes for which they are marketed [28]. To add to this concern, many patients do not report uses of NHPs to their health care providers and are less likely to attribute adverse events to NHPs, due to the belief that NHPs are inherently safe [5,31–36].

The foregoing discussion highlights significant concerns surrounding the marketing, licensing and use of NHPs in Canada. These concerns will likely have an impact on the views and practices of health care professionals who are involved in the marketing of NHPs and who are expected to provide guidance on the use of such products to consumers. Given that a majority of NHPs offered for sale are purchased at a community pharmacy [37], pharmacists, more than most other health care professionals, are likely to be involved in the marketing and provision of NHPs. In Alberta, pharmacists are obligated by applicable standards of practice to assist pharmacy patrons with choosing NHPs and to provide counselling regarding safe and effective uses of NHPs [38]. However, to date, the ways in which Canadian pharmacists accomplish these roles and the degree to which concerns over the safety, efficacy, marketing and regulation of NHPs are addressed in Canadian pharmacy care have not been studied.

The objective of this study is to assess the attitudes and practices of Alberta pharmacists regarding NHPs offered for sale in community pharmacies. The study also explores Alberta pharmacists’ views of the evidentiary basis for clinical and related uses of NHPs, the indications for which NHPs are used and the regulatory process for approval and licensing of NHPs. The study aims to provide insights on the involvement of Alberta pharmacists in pharmacy care relating to NHPs, to highlight valuable or detrimental practices, and to produce information that may help shape policy and practice in relation to NHPs.

## Methods

This study is based on a survey conducted on Qualtrics, a web-based data collection and analysis software. The sample population consisted of 4003 licensed pharmacists in the Canadian province of Alberta who had indicated to the Alberta College of Pharmacists that they were willing to be contacted for research purposes. As of January 1, 2016, there were 4,896 licensed pharmacists in Alberta [39]. The sample population represents eighty-one percent (81%) of the total number of licensed pharmacists in Alberta. Data collection began on July 16, 2015. Emails with a link to the survey were sent via Qualtrics to the pharmacists in the sample population. Three emails failed or bounced, resulting in a sample of 4000 potential participants. Three reminder emails were sent out at weekly intervals, including a final reminder sent on August 10, 2015. The survey was closed on August 13, 2015, mainly due to a sharp drop in the response rate. A total of 403 pharmacists, amounting to ten percent (10%) of the sample population, participated in the survey. The number of participants represents eight percent (8%) of the total number of licensed pharmacists in Alberta.

The study instrument consisted of 15 structured questions designed using a combination of open-ended, closed and rating scale questions (see S1 File for the study questionnaire). Six questions in the survey requested general demographic information along with information on participants’ practice setting, experience and qualifications. The remaining nine questions sought to gather data on the role of pharmacists in recommending NHPs sold in community pharmacies, commonly recommended NHPs, associated indications, and counselling practices. Three of the 15 questions in the survey allowed for open-ended responses (i.e. “Other–please specify”). The study instrument was reviewed for structure and consistency with the study objectives by an assessment specialist in the Faculty of Pharmacy and Pharmaceutical Sciences, University of Alberta. The study was also reviewed and approved by the University of Alberta Research Ethics Board. A letter of invitation to participate in the study was sent by email to the 4003 pharmacists in our initial sample. The letter informed the participants that consent to participate in the study will be implied with completion of the online survey.

Survey responses were analyzed in Qualtrics. Reported statistical calculations were automatically generated in Qualtrics. To allow for a more focused analysis, only p-values less than or equal to 0.05 are reported. Open-ended responses were analyzed manually and independently and are reported as a subset of the main category (i.e. “Other–please specify”) that elicited the responses (the complete results database is included in S2 and S3 Files).

## Results

### Question response rate

Of the 403 participants to the survey (403/4000 = ~10%), 396 (98%) completed the entire survey. Six participants completed 90% of the survey, while the remaining participant completed 80% of the survey. The lowest number of responses recorded per question was 399. A majority of the participants (365; 91%) completed the survey in 10 minutes, while the others spent between 20 and 150 minutes to complete it.

### Qualifications, practice setting and NHP-related training

Of the 403 survey participants, 300 (75%) indicated that their practice setting was in an urban area, while 102 (25%) identified as rural pharmacists. One participant did not indicate a practice setting. Number of years spent in practice ranged from zero (or less than one) to 46 years and the average number of years spent in practice was 14.4 years. Number of hours spent weekly providing direct patient care ranged from zero to 160, with an average of 33 hours worked per week. Six of the participants had no direct patient care experience, while a majority of participants (293; 73%) spent between 30–60 hours a week providing direct patient care.

To determine the participants’ qualifications and scope of practice, the survey asked them to indicate the highest degree completed and whether they have obtained additional authorization to initiate and manage drug therapy (i.e. additional prescribing authorization). A higher proportion of participants (351; 87%) indicated that a Bachelor’s degree was the highest degree completed followed by those who have completed a Master’s degree (19; 5%), Post-Baccalaureate Doctor of Pharmacy (Post Bac Pharm D) (10; 2%) and doctorate (PhD) (6; 1%). One participant selected the entry level Doctor of Pharmacy (entry level Pharm D) as the highest degree completed and the remaining 15 participants (4%) selected the “other” category. Among the latter group, five indicated that they have obtained the Accredited Canadian Pharmacy Residency designation, four have completed hospital residencies, two have completed Master’s degrees with post graduate certificates in Clinical Studies, one participant had attained two Bachelor’s degrees and one was a certified respiratory, diabetes and tobacco educator. Two of the 15 participants that selected the “other” category did not specify highest degree completed. Only 98 participants (24%) have obtained additional prescribing authorization; the majority of participants (302; 75%) did not have this authorization.

Next, the survey sought to determine whether the participants have received training relating or specific to NHPs by asking them to indicate the number of hours spent on accredited or non-accredited learning on NHPs. A majority of the participants (343; 85%) indicated that they have spent time on NHP-related learning, while 58 participants (14%) indicated that they have not spent any time learning about NHPs. Among those who have received some training, 135 (39%) indicated they have spent one to three hours on accredited and non-accredited NHP learning, 107 (31%) have spent more than 10 hours, 78 (23%) have spent four to six hours and 23 (7%) have spent seven to 10 hours.

### Recommendations relating to NHPs

The survey explored recommendations of NHPs made by community pharmacists through a series of questions regarding the most frequently recommended NHPs, the primary basis for recommending NHPs (or the source of the recommendations), frequency of recommendations and indications that NHPs are commonly recommended for. The questions in this part also probed whether participants recommend NHPs that do not have a Health Canada product number and whether participants recommend that their clients use NHPs concurrently with and/or as an alternative to conventional medicines.

#### Frequency of recommendations

The survey asked participants to rate how regularly they recommend NHPs to clients on a scale of 1 to 5 with 5 being very often, 4 being often, 3 being sometimes, 2 being rarely and 1 being never. A majority of participants (182; 45%) selected sometimes followed by those who rarely recommend NHPs (114; 28%). Among the remaining participants, 71 (18%) recommend NHPs often, 23 (6%) make recommendations very often and 11 (3%) reported that they never recommend NHPs to their clients.

#### Frequency of recommendations of specific NHPs

Vitamin D was selected as the most frequently recommended NHP (selected by 326 or 81% of the participants). Other most frequently recommended NHPs include calcium (310; 77%), multivitamins and prenatal vitamins (287; 71%), probiotics (265; 66%), fish oil and omega-3 fatty acids (237; 59%), iron (221; 55%), melatonin (212; 53%) and Vitamin B (177; 44%). The least recommended NHP categories were St. John’s wort (*Hypericum perforatum*) (12; 3%), weight loss and detoxification products (18; 4%), gingko biloba (20; 5%), ginseng (21; 5%) (Note: There are multiple species of ginseng. We did not specify ginseng species in the study questionnaire, but the most common are *Panax ginseng* and *Panax quinquefolius*), saw palmetto (*Serenoa repens*) (26; 6%) and garlic (*Allium sativum*) (26; 6%). Homeopathic products were selected as the most frequently recommended NHPs by 28 participants (7%), while 27 participants (7%) specified other products, including “PEG powder” (Polyethylene glycol-3550), “ginger” (*Zingiber officinale*), glucosamine, co-enzyme Q10, turmeric (*Curcuma longa*), curcumin, kelp (*Laminariales*), “herbals,” Vitamin B1, soy phytoestrogens, black cohosh (*Actaea racemosa*), selenium, rosemary (*Rosmarinus officinalis*), lysine, butterbur (*Petasites hybridus*), grape seed (*Vitis vinifera*) extract, soluble fiber, alpha-lipoic acid, N-acetylcysteine, glutathione, “greens,” “cell protectors,” “immunocal,” “EMP” (essential mineral powder), rhodiola (*Rhodiola rosea*) and “adrenal support” (see Table 1 for a list of NHPs arranged by frequency of recommendations and S3 File for the full list of most frequently recommended NHPs and associated responses/frequencies).

**Table 1:** Natural health products, by frequency of recommendation.

Natural health product	Number of participants who made recommendation
**Vitamin D**	**326**
Calcium	310
**Multivitamin/prenatal vitamin**	**287**
Probiotics	265
**Fish oil/omega-3 fatty acids**	**237**
Iron	221
**Melatonin**	**212**
Vitamin B12 (oral or sublingual)	178
**Psyllium fiber**	**160**
Vitamin B complex	154
**Folic acid**	**145**
Magnesium	134
**Vitamin C**	**91**
Cranberry	89
**Zinc**	**49**
Tea tree oil	43
**Echinacea**	**39**
Vitamin B6	38
**Homeopathic products**	**28**
Other	27
**Saw palmetto (*Serenoa repens*)**	**26**
Garlic (*Allium sativum*)	26
**Ginseng**	**21**
Gingko biloba	20
**Weight loss/detoxification products**	**18**
St. John’s wort (*Hypericum perforatum*)	12

#### Indications

The indication for which NHPs are most commonly recommended is vitamin and/or mineral deficiency (354; 88%). Other top indications include bone and musculoskeletal disorders (237; 59%), maintenance of general health (230; 57%), gastrointestinal disorders (180; 45%), pregnancy (158; 39%), immune system support (138; 34%), pain (121; 30%), women’s health disorders (104; 26%), cardiovascular diseases (85; 21%), allergy prevention and treatment (59; 15%) and infectious diseases (58; 14%). The least frequent indications in the dataset include respiratory disorders (27; 7%), neurological disorders (35; 9%) and weight loss and detoxification (44; 11%). Thirty-five participants (9%) specified other indications, including sleep and insomnia, cough and cold, men’s health, stress related disorders, adrenal support, hematological conditions, fertility, insect bites and minor pain, alcohol withdrawal, osteoporosis and management of side effects of other medications (see Table 2 and S3 File for a full list of indications).

**Table 2:** Indications for which natural health products are commonly recommended.

Indication	Number of participants who selected indication
**Vitamin and/or mineral deficiency**	**354**
Bone and musculoskeletal disorders	237
**Maintenance of general health**	**230**
Gastrointestinal disorders	180
**Pregnancy**	**158**
Immune system support	138
**Pain**	**121**
Women’s health disorders	104
**Cardiovascular disorders**	**85**
Allergy prevention and treatment	59
**Infectious diseases**	**58**
Dermatological disorders	57
**Food intolerances**	**55**
Psychiatric disorders	46
**Pediatric conditions**	**45**
Metabolic/Endocrine disorders	45
**Ophthalmological disorders**	**45**
Weight loss/detoxification	44
**Neurological disorders**	**35**
Other	35
**Respiratory disorders**	**27**

#### Primary basis for recommending NHPs/source of recommendations

Review articles in the Pharmacist’s Letter and Canadian Pharmacists Journal were selected as the primary basis for recommending NHPs by a majority of the participants (140; 35%) followed by client requests (93; 23%) and primary literature (55; 14%). Fewer participants relied on Health Canada approval (48; 12%), recommendations or prescriptions from primary care providers (31; 8%) and manufacturer information (13; 3%) as the primary basis for recommending NHPs to clients. Only two participants rely on the Internet and social media as the primary basis for their recommendations. Nineteen participants (5%) specified other sources, including seminar education, private research, PubMed, continuing education events and programs, personal experience, the Compendium of Therapeutic Choices and the Natural Medicines Comprehensive Database (NMCD).

#### Natural Products Number/Homeopathic Medicines Number

A majority of the participants (233; 58%) stated that they have not recommended NHPs that do not have a NPN or DIN-HM. The remaining participants had either recommended NHPs without a NPN or DIN-HM (46; 11%) or were not sure if they had or not (122; 30%).

#### Concurrent and alternative use

A majority of participants (339; 84%) indicated that they have recommended that their clients use NHPs concurrently with conventional medicines. Forty-four participants (11%) stated that they have not recommended concurrent use, while 17 (4%) indicated that they were not sure if they had made this recommendation. Regarding alternative use, 243 participants (60%) indicated that they have not recommended NHPs as an alternative to conventional medicines, while 125 participants (31%) indicated that they have recommended alternative use. The remaining 31 participants (8%) were not sure if they had made this recommendation.

### Counselling practices relating to NHPs

The remaining two questions in the survey queried the circumstances in which participants provide counselling regarding NHPs to their clients and the source of the safety and efficacy information provided during counselling. Regarding circumstances, 363 participants (90%) indicated that they provide counselling when a client inquires about NHPs. A close number and proportion of participants (353; 88%) provide counselling when recommending NHPs to clients, while 273 participants (68%) provide counselling when a client is picking up NHPs recommended by another health care provider. The number of participants who provide counselling when a client requires assistance locating NHPs in the pharmacy was 246 (61%). Four participants (1%) stated that they have never provided counselling to clients regarding the safety or efficacy of NHPs, while 11 participants (3%) specified other circumstances (see Table 3 for a list of other circumstances).

**Table 3:** “Other” circumstances in which counselling on natural health products is provided.

If there are interactions with NHP that they are taking and other prescription medications
We have a nutritionist on site who I bring in to expand on the consultation I am able to provide
When admitted to hospital and taking NHPs at home prior
When a client is being discharged in the next week on an NHP
When combing with prescription medication
When a client appears to be questioning the Rx drug and may choose to go NHP route on own. . . depending on Diagnosis that [patient] has—and depending on the current "flavour of the day" on social media
When I have concerns regarding an interaction with another treatment
When they use concurrent prescription meds
I work in a PCN so some options don't apply

Regarding the source of safety and efficacy information provided during counselling, most participants (283; 70%) identified the NMCD as the main source of safety and efficacy information provided during counselling. Other primary sources selected by the participants include Health Canada product monographs (44; 11%), Canadian Pharmacist’s Letter (32; 8%) and the manufacturer’s product monograph (25; 6%). The fewest number of participants (16; 4%) specified other primary sources, including personal knowledge, review articles, the Compendium of Therapeutic Choices, Canadian Pharmacists Association e-Suite, Lexicomp, Medscape, Natural Standard, PubMed, science-based journals, Internet searches, the Science Based Medicine and Pharmacy Blog and “respected authors of evidence based medicine [such as] Dr. Edzard Ernst.”

### Influence of frequency of NHP recommendations on counselling practices

Among participants who recommend NHPs often or very often (n = 94), more indicated that they provide counselling when recommending NHPs to clients (86; 91%) or responding to client inquiries about NHPs (80; 85%) than when clients are picking up NHPs recommended by another health care provider (71; 76%) or requesting assistance with locating NHPs in the pharmacy (64; 68%).

### NHPs recommended for concurrent use

Participants who have recommended that their clients use NHPs concurrently with conventional medicines (n = 339) indicated that they most frequently recommend Vitamin D (283; 83%), calcium (275; 81%), multivitamins and prenatal vitamins (248; 73%) and probiotics (234; 69%) to their clients. Among this category of participants, 23 (7%) selected homeopathic products as the NHPs they recommend most often. The least frequently recommended NHPs by the participants in the concurrent use category include St. John’s wort (*Hypericum perforatum*) (11; 3%), weight loss and detoxification products (14; 4%) and gingko biloba (15; 4%).

### NHP recommendations by practice setting, source of recommendation and learning

The most frequently recommended NHPs were similar for participants in rural and urban settings. However, among participants in rural practice (n = 102), a higher proportion recommend probiotics (77%), magnesium (48%), psyllium fiber (46%), zinc (21%), garlic (*Allium sativum*) (13%), echinacea (13%) (Note: There are multiple species of echinacea. We did not specify echinacea species in the study questionnaire but the most common is *Echinacea purpurea*), saw palmetto (*Serenoa repens*) (11%), and ginseng (8%) compared to the corresponding proportion of urban practitioners (n = 300) for the same product categories, as follows: probiotics (63%), magnesium (29%), psyllium fiber (38%), zinc (9%), garlic (*Allium sativum*) (4%); echinacea (9%), saw palmetto (*Serenoa repens*) (5%), and ginseng (4%).

Participants who rely on review articles, Health Canada approval, the primary literature and recommendations from other primary care providers as the primary basis for recommending NHPs to clients selected Vitamin D as the most frequently recommended NHP. Calcium was the most frequent recommendation for those who rely on client requests, while those who rely primarily on manufacturer’s information favour multivitamins and prenatal vitamins. A higher proportion of participants who rely on Health Canada approval (21%) and review articles (21%) selected homeopathic products as the most frequently recommended NHPs compared to participants who rely on manufacturer’s information (14%), the primary literature (11%) and client requests (14%).

The number of hours spent on accredited and non-accredited NHP-related learning did not alter observed recommendation trends. Vitamin D and calcium remained the top recommendations when this variable was introduced. Among participants who selected homeopathic products as the most frequently recommended NHPs (n = 28), 11 (39%) have spent greater than 10 hours on accredited or non-accredited NHP learning.

## Discussion

### Prevalence of NHP practice among pharmacists

Our findings indicate that pharmacy care relating to NHPs is prevalent among community pharmacists in Alberta and may even be considered integral to their practice. A majority of pharmacists involved in the study (276; 68%) routinely or occasionally recommend NHPs to clients, while, conversely, very few (11; 3%) have never recommended NHPs. This finding suggests that pharmacists have embraced a role in facilitating access to NHPs, which are, for the most part, regulated health products. This role may include helping consumers understand the evidence base for NHPs. Conversely, by undertaking this role, pharmacists may also be facilitating public demand for products that lack good quality or well-established evidence to support safe or effective uses [8–22]. Such involvement in facilitating access may, in turn, undercut the view that pharmacy is an evidence-based profession practiced by professionals who are trained to administer products that have been rigorously and scientifically tested to ensure that they are safe and effective for public use. These findings also raise questions about the extent to which pharmacists should be involved in the marketing of NHPs and about the proper role of pharmacists in community pharmacy settings where products other than pharmaceuticals are offered to the public [40–44].

Given that most, if not all, NHPs are marketed to the public over-the-counter [45], the involvement of pharmacists in initiating or facilitating access to NHPs may serve as clinical endorsements of NHPs and may convey the impression to the public that these products are generally clinically safe or effective for recommended uses. While solid clinical evidence may exist for some NHPs that warrant such practices, the evidence around a large number of NHPs remains understudied and unclear. This concern is mediated somewhat by the finding that a majority of pharmacists involved in the study provide counselling when recommending, dispensing or dealing with client inquiries about NHPs. However, it is unclear from our findings if such counselling addresses the evidentiary questions surrounding NHPs or if it serves as a deterrent to demand. While likely not the intention, the routine provision of counselling may serve to simply increase public confidence in NHPs.

The foregoing observations are consistent with findings of other studies dealing with attitudes and practices of community pharmacists regarding NHPs [46–47]. The pharmacists surveyed in these studies reported that consumers routinely requested information and advice on NHPs from them. The studies also found that although the pharmacists surveyed are generally open to NHPs, they perceive their role as focused mainly on ensuring patient safety by providing counselling and improving their training on NHPs [46–47]. One study found that the majority of customers who inquire about NHPs make product purchases [46] and that pharmacists are motivated to meet increased demand for NHPs by “stock[ing] more products” [46].

### Impact of regulatory approval on recommendations of NHPs

A majority of participants in this study (233; 58%) stated that they only recommend NHPs that have a Health Canada product number to clients. While this finding indicates regulatory compliance, it raises serious concerns in light of the flaws in the licensing system and regulatory process. As discussed earlier, NHPs are subject to less rigorous pre-approval scrutiny and evidentiary demands compared to conventional drugs. Under applicable regulations, persons seeking to license NHPs are required to provide “information that supports the safety and efficacy of the natural health product when it is used in accordance with the recommended conditions of use” [48]. However, unlike conventional drugs, the supporting information need not be comprised of preclinical or clinical trial data showing that the product is safe or efficacious. Rather, pre-market review of NHPs is based on a system of risk assessment that considers the “level of certainty” associated with the product’s “established safety and efficacy profile” [49]. NHPs that have a high level of certainty, i.e. a “well-established safety and efficacy profile,” face less scrutiny that those that have a medium or low level of certainty. Health Canada regulations and policies are generally unclear on what constitutes a “well-established safety and efficacy profile,” but will accept a variety of forms of supporting evidence depending on the product’s certainty profile and intended use, including Phase I—III randomized controlled trials, meta-analysis, observational studies, prospective and retrospective studies, evidence of decision from another regulatory agency, systematic reviews, narrative reviews that cite primary evidence, epidemiological studies, published compilations referring to traditional use, pilot and open label studies, reputable textbooks and product demonstrations [49]. The diversity of options for supporting evidence works favourably for the introduction of NHPs that are not evidence-based into the market [26].

The finding that a majority of recommendations are based on the problematic licensing system is somewhat attenuated by the fact that a low number of participants (48; 12%) rely on Health Canada approval as the primary or sole basis for recommending NHPs. However, a noteworthy number/proportion of participants are either unsure that they have recommended licensed NHPs (122; 30%), or have recommended unlicensed products to clients (46; 11%). Both findings indicate non-compliance with or lack of knowledge of regulatory requirements and suggest a need for education on licensing and regulatory requirements for NHPs.

### Reliance on the Natural Medicines Comprehensive Database (NMCD)

The NMCD is a reputable resource that employs an evidence-based approach to assigning effectiveness and safety ratings to NHPs for specific indications [50]. The NMCD is the Pharmacy Examining Board of Canada approved resource for NHPs. As such, it is logical and appropriate that most pharmacists in this study (283; 70%) identified it as their primary resource relied on when counselling pharmacy patrons about NHPs. Conversely, it is concerning that only three participants in the study rely on the NMCD as the primary basis for recommending NHPs to clients.

The effectiveness ratings provided by the NMCD are based on the quantity and quality of available evidence. The NMCD advises that only NHPs with the combined ratings of “effective” and “likely safe” or “likely effective” and “likely safe” should be recommended to patients [51]. NHPs are rated as “effective” if they have clinical evidence of effectiveness for a specific indication that is consistent with or equivalent to passing a regulatory review by the FDA, Health Canada or equivalent body and are supported by two or more high quality non-biased randomized clinical trials or meta-analyses including several hundred to several thousand patients. A rating of “likely effective” means that the NHP meets the same criteria as “effective” but has not passed a regulatory review and is supported by clinical trials involving several hundred patients. Safety ratings in the NMCD are based on similar criteria. It should be noted that in this study, we did not seek to explore or establish how pharmacists interpret the safety and effectiveness ratings in the NMCD or how they apply such ratings in the clinical context. It is also unclear from our study whether pharmacists recommend NHPs that do not meet the combined ratings endorsed by the NMCD. However, our study does suggest the need for further research exploring these matters, particularly in light of the finding that a majority of pharmacists in our study rely on the NMCD when counselling clients about NHPs.

One concern that emerges from the finding that pharmacists rely on the NMCD for counselling on NHPs surrounds the reliability of the rating system. The use of regulatory approvals in assigning ratings is particularly problematic given the questions raised earlier around the rigor of such approvals. Our examination of specific product ratings in the NMCD also revealed some inconsistencies between the stated rating and the evidence used to support the rating. For example, calcium (one of the top NHPs recommended by pharmacists in our study) is rated in the NMCD as “effective” for the reversal of hyperkalemia when administered intravenously [52]. However, a review of the studies used to support the rating makes it clear that calcium does not reverse hyperkalemia and is only effective for the treatment of ECG changes that may occur due to hyperkalemia [53–55]. Also, although calcium is listed as FDA-approved for hyperkalemia [52], it is not clear if the “effective” rating is merely based on such approval or on the supporting studies, which are mainly review articles and summaries of the evidence. Regardless, the rating appears to be inaccurate with regard to the specific indication and could result in misleading recommendations and incorrect clinical uses of calcium.

### Recommendations and clinical benefit

This study did not examine indications for which specific NHPs are recommended or specific categories of patients to whom recommendations are made. While the questions regarding NHP recommendations and indications allowed for open-ended responses, we should caution that there is no way to determine from the study results whether the NHP recommendations by the study participants are correlated with the reported indications. Thus, our findings should not be interpreted as establishing that the study participants recommend any of the listed or identified NHPs for any of the listed or identified indications. However, given the open-ended nature of the responses to the questions regarding NHP recommendations and indications, it is worth asking if there is any credible evidence of significant clinical benefit from using the recommended NHPs to treat or address indications listed or identified in the study findings. Our limited review of the literature suggests that available evidence of clinical benefit from the recommended NHPs is generally low or of substandard quality, or based on studies plagued with issues of poor design, publication bias and limited participant enrolment [56–59]. Most of the NHPs included in this study have not been found to provide any clinical benefits in randomized, double-blind clinical studies. Vitamin D, the most recommended NHP in this study, has been shown to provide clinical benefit when used to treat vitamin deficiencies, but there is little to no evidence that it provides any clinical benefits when used to treat other conditions [60]. Calcium, another commonly recommended NHP, has been shown to reduce the risk of early onset pre-eclampsia, but only in women with low calcium intake [56,61]. However, a recent review of clinical studies of calcium supplementation for the prevention of pre-eclampsia concluded that the utility of the supporting evidence is limited due to issues with trial design and publication bias [56]. Based on our findings, we recommend that further research be undertaken to assess the clinical uses and benefits of the recommended NHPs in the study findings.

Pharmacists in Alberta are required under applicable standards of practice to “offer assistance to… patient[s] who wish[] to purchase…health care product[s],” including NHPs (see Standard 9) [38] and our findings confirm compliance with this requirement. However, issues surrounding the credibility and utility of supporting evidence for clinical recommendations of the NHPs included in our study raise questions regarding the role that pharmacists should play in the marketing of such products, all of which are sold over-the-counter in community pharmacies. The involvement of pharmacists in marketing NHPs may serve to legitimize clinical applications of products that may not provide clinical benefits for the indications they purport to treat. While it is possible that the actual involvement of pharmacists is limited to informing patients about the available evidence for clinical uses of specific NHPs or about safe use of NHPs, our findings suggest that our participants’ roles extend to initiating the clinical encounter and making recommendations.

### Inappropriate and anomalous practices around NHPs

A very small number of pharmacists involved in the study (28; 7% of participants) selected homeopathic products as their most frequently recommended NHPs. While this finding cannot be interpreted as demonstrating any significant trends, it is extremely worrisome given that there is virtually no credible scientific evidence to support clinical recommendations of homeopathic products. Existing systematic reviews of clinical research on a variety of homeopathic remedies conclude that they offer no statistically significant treatment or health effects and that there is no evidence of efficacy for the various indications tested [62–63]. There is also a notable lack of high quality randomized controlled trials or other clinical research studies designed to assess the safety and efficacy of homeopathic remedies, and available studies are mainly poorly designed, of low quality, and unreliable [64–66].

While the specific basis for recommending homeopathic products cannot be established from our findings, it is doubtful that the recommendations are based on reputable evidence-based sources such as the Canadian Pharmacist’s Letter and peer-reviewed journal articles, which were selected by study participants as the top two sources upon which their recommendations are based. One possible source is reliance on the fact that the products have been issued a DIN-HM (i.e. a Health Canada product number). As previously discussed, a majority of pharmacists in this study (233; 58%) only recommend NHPs that have received a Health Canada product number to clients. Given that evidence or information to support recommendations of homeopathic products is either non-existent or not readily available (the NMCD, for example, does not contain any entries for homeopathic products), it is likely that pharmacists simply rely on the Health Canada “seal of approval” as a basis for recommending this category of products. As noted earlier, reliance on Health Canada product numbers as a basis for recommending NHPs is less than ideal given the issues associated with regulatory oversight and approval of NHPs and may be even worse in relation to homeopathic products.

Another factor that may be driving recommendations is a trend towards aggressive marketing of homeopathic products by community pharmacies. Most major Canadian pharmacy chains currently have dedicated product displays for homeopathic and naturopathic remedies, often times located next to the pharmacy. The location and availability of these products, combined with client inquiries and requests, may have some influence in driving recommendations by pharmacists.

A related and potentially problematic finding is that participants who selected homeopathic products as their top recommendation have also spent ten hours or greater on accredited or non-accredited NHP learning. This finding suggests that learning about NHPs may not necessarily alter clinical practices around NHPs, including practices and recommendations that one might expect to be addressed by further learning, such as the lack of evidence for efficacy in relation to homeopathic remedies. Indeed, the number of hours spent on accredited and non-accredited NHP-related learning did not have any significant impacts on recommendation trends observed in the study. While no conclusions regarding the type or quality of further learning about NHPs can be drawn from these findings, the data does suggest a need to evaluate the quality of continuing education on NHPs that pharmacists receive.

### Integrative practices

The concept of integrative medicine, which seeks to combine the best evidence in both Western and complementary and alternative medicine for the delivery of optimal health care, is growing in popularity in health care and health education [67–68]. The finding that 8% of participants in our study have provided care to clients who present recommendations or prescriptions of NHPs from their primary care providers reflects this trend. Under an integrative medicine approach, NHPs with a strong research infrastructure are incorporated into therapeutic modules to help pharmacists determine what role or benefit NHPs may have alongside conventional drugs. The finding that the majority of pharmacists in this study (339; 84%) would recommend using NHPs concurrently with conventional drugs is consistent with an integrative approach. At the same time, the approach and trend suggested by this finding is concerning when considered against the lack of evidence regarding the safety and interaction potential of NHPs. Such interactions can be antagonistic or synergistic in nature, but clinical interpretation is still needed in both cases. While some NHPs, such as St. John’s wort (*Hypericum perforatum*), have a reasonably well-established safety profile [69], credible clinical evidence on the safety profiles of most NHPs is missing or unclear. Pharmacists who recommend concurrent use of NHPs and conventional drugs may therefore be exposing their clients to safety risks associated with drug-NHP interactions.

The finding that 125 participants (31%) recommend NHPs as an alternative to conventional drugs is also consistent with this integrative approach. However, such recommendations pose potential risks to clients who may forgo proven treatments in favour of NHPs that do not have an established safety or efficacy profile, including the risk of not receiving timely or appropriate treatment. The latter risk is highlighted or illustrated by a recent case involving an Alberta toddler who died of bacterial meningitis after the parents opted to forgo medical treatment with antibiotics in favour of treating him with NHPs, including echinacea, one of the NHPs included in our study [70–71]. Recommendations of alternative use may also be inconsistent with the pharmacist’s expertise and scope of practice. Pharmacists presently do not receive any significant training in the pharmacologic, pharmacokinetic and safety profiles of NHPs and it is doubtful that they possess sufficient expertise and information to recommend NHPs in place of conventional drugs. Also, it is not clear to what extent such alternative use recommendations are backed by full patient histories related to NHP use, safety data around NHPs and potential interactions with conventional drugs. These findings, at a minimum, suggest a need to evaluate the role of pharmacists in the context of integrative approaches to health care. They further suggest that it is imperative that pharmacists receive training and continuing education on existing and emerging clinical trends and evidence on NHPs. The standards of pharmacy practice in Alberta currently do not specifically require such training or continuing education. As such, the development of NHP-focused continuing education policies should be made a priority, as this will help pharmacists make appropriate and safe recommendations of NHPs.

## Study Limitations

Given the cross-sectional nature of this study, we are unable to assume or determine the factors driving the practices reported by our participants, or to offer further interpretation. Further research exploring pharmacists’ beliefs and attitudes regarding specific NHPs will be valuable and will further understanding of clinical trends and issues surrounding pharmacy care relating to NHPs.

Given the study design, respondent bias is likely to have played a role as those who chose to respond to the study may be more interested in NHPs or more likely to recommend them in their practice setting. While we were able to capture eight percent (8%) of licensed pharmacists (403/4896) in the province of Alberta in Canada through this survey, other important differences may exist that we are unable to generalize from our study sample.

## Conclusion

The growing consumer demand for NHPs implicates pharmacy practice in certain fundamental respects. NHPs are typically marketed or sold in community pharmacies and pharmacists, by virtue of their “front line” role in the pharmacy setting, are both expected and required by their professional practice guidelines to provide care relating to NHPs to their clients. This study sought to establish the views and practices of pharmacists in Alberta in regards to NHPs offered to the public in community pharmacies. The study findings indicate a high prevalence of pharmacy care relating to NHPs among study participants. Although pharmacists’ practices around NHPs are consistent with the existing licensing framework, we found some involvement in problematic practices that necessitate further research and potential policy scrutiny.

## Supporting Information

S1 File Study questionnaire. (PDF)

S2 File Study results–frequencies. (PDF)

S3 File Study results–cross tabulations. (PDF)
