# Supplementary material for: Community Pharmacists’ Views and Practices Regarding Natural Health Products Sold in Community Pharmacies
Source: PLoS One. 2016 Sep 23;11(9):e0163450. doi: 10.1371/journal.pone.0163450 (PMC5035072; doi:10.1371/journal.pone.0163450)
Supplement: S1 File — (PDF) [file pone.0163450.s001.pdf]

S1 File. Study question naire

Survey Questions

The purpose of this survey is to collect information on community pharmacists' views and practices around Natural Health Products (NHPs) in Alberta.

NHPs include:

- **Vitamins and minerals**
- **Herbal remedies**
- **Homeopathic medicines**
- **Traditional medicines, such as traditional Chinese medicines**
- **Probiotics**
- **Amino acids and essential fatty acids**

NOTE: Substances that are administered by puncturing the dermis (e.g. subcutaneous vitamin B12 injections) are NOT considered NHPs under applicable government regulations.

The survey will take 5 - 10 minutes to complete.

Your participation in the survey is anonymous and confidential.

Thank you for taking the time to complete the survey.

Please indicate your practice setting.

- Rural
- Urban

How many years have you been in practice?

Please indicate the number of hours per week you work providing direct patient care:

Please indicate the highest degree you have completed:

- Bachelor's Degree
- Master's Degree
- PhD
- Entry Level PharmD
- Post-Baccalaureate PharmD
- Other (please specify):

Please indicate whether you have Additional Prescribing Authorization (APA):

- Yes
- No

Please indicate the number of hours you have spent on accredited and non-accredited NHP learning within the past two years:

- None
- 1 to 3 hours
- 4 to 6 hours
- 7 to 10 hours
- More than 10 hours

On a scale of 1 to 5, with 5 being very often and 1 being never, how regularly do you recommend NHPs to clients?

- 1- Never
- 2-Rarely
- 3-Sometimes
- 4-Often
- 5-Very Often

What is your primary basis for recommending NHPs to clients? Check one.

- Health Canada approval of NHP
- Manufacturer information
-

- Primary literature
- Review articles (i.e. Pharmacist’s Letter, CPJ)
- Client request
- Recommendation/prescription from primary care provider
- Internet/social media
- Other (please specify):

What indications do you commonly recommend NHPs for? Check all that apply.

- Maintenance of general health
- Vitamin and/or mineral deficiency
- Women’s health disorders
- Pregnancy
- Bone and musculoskeletal disorders
- Psychiatric disorders
- Neurological disorders
- Pain
- Immune system support
- Allergy prevention and treatment
- Food intolerances
- Cardiovascular disorders
- Metabolic/Endocrine disorders
- Gastrointestinal disorders
- Infectious diseases
- Respiratory disorders
- Dermatological disorders
- Ophthalmological disorders
- Pediatric conditions
- Weight loss/detoxification
- Other (please specify):

What NHPs do you recommend most often? Check all that apply.

- Multivitamin/prenatal vitamin
- Vitamin B complex
- Vitamin B6

- Vitamin B12 (oral or sublingual)
- Folic acid
- Vitamin C
- Vitamin D
- Calcium
- Magnesium
- Zinc
- Fish oil/omega-3 fatty acids
- Melatonin
- Iron
- Ginseng
- Echinacea
- Homeopathic products
- Probiotics
- St. John's wort
- Cranberry
- Garlic
- Gingko biloba
- Tea tree oil
- Saw palmetto
- Psyllium fiber
- Weight loss/detoxification products
- Other (please specify): ____________

Have you recommended NHPs that do not have a Natural Products Number (NPN) or Homeopathic Medicine Number (DIN-HM) to clients?

- Yes
- No
- Not Sure

Have you recommended NHPs as an alternative to conventional medicines?

- Yes
- No
- Not Sure

Have you recommended that a client use NHPs concurrently with conventional medicines?

- Yes
- No
- Not Sure

In what circumstances do you provide counselling to clients regarding the safety and efficacy of NHPs? Check all that apply.

- When I recommend a NHP to a client
- When a client inquires about a NHP
- When a client is picking up a NHP prescribed or recommended by another health care provider
- When a client requires assistance locating a NHP in the pharmacy
- I have never provided counselling to clients regarding the safety and efficacy of NHPs
- Other (please specify):

What is the primary source of the safety and efficacy information that you provide during counselling? Check one.

- Health Canada product monograph
- Manufacturer product monograph
- Natural Medicines database
- Canadian Pharmacist's Letter
- Other (please specify):
