# Supplementary material for: Community Pharmacists’ Views and Practices Regarding Natural Health Products Sold in Community Pharmacies
Source: PLoS One. 2016 Sep 23;11(9):e0163450. doi: 10.1371/journal.pone.0163450 (PMC5035072; doi:10.1371/journal.pone.0163450)
Supplement: S2 File — (PDF) [file pone.0163450.s002.pdf]

Community pharmacists’ views and practices regarding natural health products sold in community pharmacies

Study results

General note: Percentages in the paper are reported as proportions of the total number of participants in the survey (403). The raw results shown below express percentages as proportions of participants who responded to each specific question.

1. Please indicate your practice setting.

#	Answer		Response	%
1	Rural		102	25.37%
2	Urban		300	74.63%
	Total		402	100.00%

Statistic	Value
Min Value	1
Max Value	2
Mean	1.75
Variance	0.19
Standard Deviation	0.44
Total Responses	402

2. How many years have you been in practice?

Text Response
Refer to **Appendix A, below** for full results.

Statistic	Value
Total Responses	399

3. Please indicate the number of hours per week you work providing direct patient care:

Text Response
Refer to **Appendix B, below,** for full results.

Statistic	Value
Total Responses	399

4. Please indicate the highest degree you have completed:

#	Answer		Response	%
1	Bachelor's Degree		351	87.31%
2	Master's Degree		19	4.73%
3	PhD		6	1.49%
4	Entry Level PharmD		1	0.25%
5	Post-Baccalaureate PharmD		10	2.49%
6	Other (please specify):		15	3.73%
	Total		402	100.00%

Other (please specify):
Hospital Residency
Master’s plus Post Graduate Certificate in Clinical Studies
Two bachelor degrees
Residency
Accredited Canadian Pharmacy Residency (ACPR)
Hospital Residency
ACPR
ACPR
Residency
ACPR
Masters plus Post Graduate Certificate in Clinical Studies
ACPR
Certified Respiratory, Diabetes & Tobacco Educator

Statistic	Value
Min Value	1
Max Value	6
Mean	1.37
Variance	1.33
Standard Deviation	1.15
Total Responses	402

5. Please indicate whether you have Additional Prescribing Authorization (APA):

#	Answer		Response	%
1	Yes		98	24.50%
2	No		302	75.50%
	Total		400	100.00%

Statistic	Value
Min Value	1
Max Value	2
Mean	1.76
Variance	0.19
Standard Deviation	0.43
Total Responses	400

6. Please indicate the number of hours you have spent on accredited and non-accredited NHP learning within the past two years:

#	Answer		Response	%
1	None		58	14.46%
2	1 to 3 hours		135	33.67%
3	4 to 6 hours		78	19.45%
4	7 to 10 hours		23	5.74%
5	More than 10 hours		107	26.68%
	Total		401	100.00%

Statistic	Value
Min Value	1
Max Value	5
Mean	2.97
Variance	2.04
Standard Deviation	1.43
Total Responses	401

7. On a scale of 1 to 5, with 5 being very often and 1 being never, how regularly do you recommend NHPs to clients?

#	Answer		Response	%
1	1- Never		11	2.74%
2	2-Rarely		114	28.43%
3	3-Sometimes		182	45.39%
4	4-Often		71	17.71%
5	5-Very Often		23	5.74%
	Total		401	100.00%

Statistic	Value
Min Value	1
Max Value	5
Mean	2.95
Variance	0.80
Standard Deviation	0.89
Total Responses	401

8. What is your primary basis for recommending NHPs to clients? Check one.

#	Answer		Response	%
1	Health Canada approval of NHP		48	11.97%
2	Manufacturer information		13	3.24%
3	Primary literature		55	13.72%
4	Review articles (i.e. Pharmacist's Letter, CPJ)		140	34.91%
5	Client request		93	23.19%
6	Recommendation/prescription from primary care provider		31	7.73%
7	Internet/social media		2	0.50%
8	Other (please specify):		19	4.74%
	Total		401	100.00%

Other (please specify):
Through seminar education and private research online
Treatment needed as determined by patient assessment
Natural Medicines Comprehensive Database
Information
Part of ideal protein weight loss method
Health Canada recommendations re: vit D/calcium. Other than that, I may recommend 'rarely'
Assessed need for supplementation
Combination of reading, conferences, etc
My own research
Pubmed
Not on hospital formulary
Research from manufacturer info and other sources
Natural Medicines Comprehensive Database
Best medication for indication presented at the time
Evidence Based Medicine Only
I took a Holistic Nutrition Course in the 1990's
CE events and programs
personal experience over the years
Therapeutic Choices, Natural Medicines Database, CEU's

Statistic	Value
Min Value	1
Max Value	8
Mean	4.03
Variance	2.69
Standard Deviation	1.64
Total Responses	401

9. What indications do you commonly recommend NHPs for? Check all that apply.

#	Answer		Response	%
1	Maintenance of general health		230	57.64%
2	Vitamin and/or mineral deficiency		354	88.72%
3	Women's health disorders		104	26.07%
4	Pregnancy		158	39.60%
5	Bone and musculoskeletal disorders		237	59.40%
6	Psychiatric disorders		46	11.53%
7	Neurological disorders		35	8.77%
8	Pain		121	30.33%
9	Immune system support		138	34.59%
10	Allergy prevention and treatment		59	14.79%
11	Food intolerances		55	13.78%
12	Cardiovascular disorders		85	21.30%
13	Metabolic/Endocrine disorders		45	11.28%
14	Gastrointestinal disorders		180	45.11%
15	Infectious diseases		58	14.54%
16	Respiratory disorders		27	6.77%
17	Dermatological disorders		57	14.29%
18	Ophthalmological disorders		45	11.28%
19	Pediatric conditions		45	11.28%
20	Weight loss/detoxification		44	11.03%
21	Other (please specify):		35	8.77%

- *continued on next page*

Other (please specify):
Rarely recommend
Osteoporosis
Sleep/insomnia
Help with side effects from other medications (i.e. Coenzyme q10)
Adrenal support
Insomnia, arthritis
Sleep
Sleep
Sound care
Insomnia
Alcohol withdrawal vitamin deficiency
Sleep aid
Probiotics with clindamycin
At Script Pharmacy we provide an integrative approach to responsible health decissions
Sleep
Not applicable
Sleep
Topical for insect bites and minor pain
Sleep
Sleep
Laxatives
Melatonin for insomnia
Children's cough
Only if lab results show deficiency and in pregnancy of maternity vitamins
Sleep
For a condition where there is a perception that "no" harm could be done with consumption/use of the product
Fertility
Cardiovascular
Hematological
Cough and cold remedies
Cancer prevention and also chemo adrs, thyroid support, memory, autism , adhd, energy, insomnia,
Usually do not recommend
Men's health, stress related disorders, adrenal support
Sleep
Insomnia

Statistic	Value
Min Value	1
Max Value	21
Total Responses	399

10. What NHPs do you recommend most often? Check all that apply.

#	Answer		Response	%
1	Multivitamin/prenatal vitamin		287	71.93%
2	Vitamin B complex		154	38.60%
3	Vitamin B6		38	9.52%
4	Vitamin B12 (oral or sublingual)		178	44.61%
5	Folic acid		145	36.34%
6	Vitamin C		91	22.81%
7	Vitamin D		326	81.70%
8	Calcium		310	77.69%
9	Magnesium		134	33.58%
10	Zinc		49	12.28%
11	Fish oil/omega-3 fatty acids		237	59.40%
12	Melatonin		212	53.13%
13	Iron		221	55.39%
14	Ginseng		21	5.26%
15	Echinacea		39	9.77%
16	Homeopathic products		28	7.02%
17	Probiotics		265	66.42%
18	St. John's wort		12	3.01%
19	Cranberry		89	22.31%
20	Garlic		26	6.52%
21	Gingko biloba		20	5.01%
22	Tea tree oil		43	10.78%
23	Saw palmetto		26	6.52%
24	Psyllium fiber		160	40.10%
25	Weight loss/detoxification products		18	4.51%
26	Other (please specify):		27	6.77%

- *continued on next page*

Other (please specify):
Probiotics in cases of antbx induced diarrhea
Probiotic
Rarely recommend
PEG powder
Ginger
Metagenics products
Glucosamine
Co enzyme q10
Turmeric, circumin, kelp, co-enzyme Q-10
Coenzyme Q10
Herbals
Vitamin B1
Calcium with other minerals for OP
None
Soy phytoestrogens, black cohosh
Selenium, rosemary,
I almost never recommend unless true deficiency which in Canada is rare
Glucosamine
Lysine (cold sores)
Butterbur
Glucosamine
Grape seed extract
Soluble fiber, co-enzyme q10, turmeric
Ala, nac, glutathione, greens, cell protectors, immunocal, EMP, rhodiola or adrenal support, selenium,
Coq10, Curcumin
Glucosamine
Alpha lipoic acid

Statistic	Value
Min Value	1
Max Value	26
Total Responses	399

11. Have you recommended NHPs that do not have a Natural Products Number (NPN) or Homeopathic Medicine Number (DIN-HM) to clients?

#	Answer		Response	%
1	Yes		46	11.47%
2	No		233	58.10%
3	Not Sure		122	30.42%
	Total		401	100.00%

Statistic	Value
Min Value	1
Max Value	3
Mean	2.19
Variance	0.38
Standard Deviation	0.62
Total Responses	401

12. Have you recommended that a client use NHPs concurrently with conventional medicines?

#	Answer		Response	%
1	Yes		339	84.75%
2	No		44	11.00%
3	Not Sure		17	4.25%
	Total		400	100.00%

Statistic	Value
Min Value	1
Max Value	3
Mean	1.20
Variance	0.24
Standard Deviation	0.49
Total Responses	400

13. Have you recommended NHPs as an alternative to conventional medicines?

#	Answer		Response	%
1	Yes		125	31.33%
2	No		243	60.90%
3	Not Sure		31	7.77%
	Total		399	100.00%

Statistic	Value
Min Value	1
Max Value	3
Mean	1.76
Variance	0.34
Standard Deviation	0.58
Total Responses	399

14. In what circumstances do you provide counselling to clients regarding the safety and efficacy of NHPs? Check all that apply.

#	Answer		Response	%
1	When I recommend a NHP to a client		353	87.81%
2	When a client inquires about a NHP		363	90.30%
3	When a client is picking up a NHP prescribed or recommended by another health care provider		273	67.91%
4	When a client requires assistance locating a NHP in the pharmacy		246	61.19%
5	I have never provided counselling to clients regarding the safety and efficacy of NHPs		4	1.00%
6	Other (please specify):		11	2.74%

- *continued on next page*

Other (please specify):
6
If there are interactions with NHP that they are taking and other prescription medications
We have a nutritionist on site who I bring in to expand on the consultation I am able to provide
When admitted to hospital and taking nhps at home prior
When a client is being discharged in the next week on an NHP.
When combing with prescription medication
When a client appears to be questioning the Rx drug and may choose to go NHP route on own... Depending on Diagnosis that pt has - and depending on the current "flavour of the day" on social media
When I have concerns regarding an interaction with another treatment
When they use concurrent prescription meds
I work in a PCN so some options don't apply

Statistic	Value
Min Value	1
Max Value	6
Total Responses	402

15. What is the primary source of the safety and efficacy information that you provide during counselling? Check one.

#	Answer		Response	%
1	Health Canada product monograph		44	11.00%
2	Manufacturer product monograph		25	6.25%
3	Natural Medicines database		283	70.75%
4	Canadian Pharmacist’s Letter		32	8.00%
5	Other (please specify):		16	4.00%
	Total		400	100.00%

- *continued on next page*

Other (please specify):
Personal knowledge
Review articles
Therapeutic choices and e-cps
Nutritionists knowledge and information he provided
Lexicomp
Manufacturer info (depends on company -some are better than others)<br>Lexicomp patient handouts
primary literature
Medscape
Science based journals...internet search
Natural Standard
Lexi-comp
Science Based Medicine & Pharmacy Blog; respected authors of evidence based medicine (i.e. Dr. Edzard Ernst)
Pubmed

Statistic	Value
Min Value	1
Max Value	5
Mean	2.88
Variance	0.73
Standard Deviation	0.85
Total Responses	400

Appendix A

How many years have you been in practice?

4
4
1
3
8
2
4
32
38
40
4
7
22
24
10
30
20
2
12
1
1
9
10
4
19
28
25
4
16
4
1
20
5
16
3
34
5
2
32
12
25

17
8
40
30
12
1
42
34
5
35
15
22
15
3
4
3
20
33
1
11
30
30
3
10

11
3
19
3
34
2
7
16
11
24
3
29
25
19
11
33
24

15
15
17
1
26
8
29
1
14
14
25
3
25
28
6
5
35
37
25
10
38
32
26
15
7
27
37
20
21
6
35
5
13
4

20
21
7
6
28
2
1

5
9
11
4
13
42
21
7
1
30
1
30
16
4
10
11
16
8
27
6
20
32
3
23
25
38
22
19
1
1
1
29
3
3
5
1
3
13
31
3
32
1

3
4
2
3
41
1
3
38
9
1
33
3
13
10
5
27
2
5
1
6
23
9
24
1
38
1
12
5
5
10
30
14
1
20
4
7
4
23
3
3
10
2

21
6
7
23
16
1
20
0
23
15
10
7
3
46
38
21
1
33
5
2
1
23
25
8
9
26
21
6
2
38
2
32
1
18
1
40
10
1
9
2
25
6

10
4
25
4
32
21
29
39
1
1
1
7
2
1
25
26
11
43
1
40
16
31
20
1
7
37
6
1
3
3
16
18
16
1
13
1
8
40
27
1
5
30

23
7
1
1
9
33
9
10
4
20
25
3
17
22
3
1
28
17
5
1
18
34
26
5
7
34
1
9
26
7
30
42
1
5
30
1
1
1
28
2
14
3

29
27
6
4
20
5
32
2
3
5
45
4
15
11
11
31
15
1
1
14
35
38
20
10
2
35
10
4
2
15
18
1
20
37
1
5
33
5
2
2
5

8
35
19
4
2
38
2
17
23
1
5
13
2
20
19
37
13
40
35
3
7
1
4
16
40

Appendix B

Please indicate the number of hours per week you work providing direct patient care:

40
40
32
40
40
60
40
35
35
40
45
8
38
16
40
0
40
40
40
35
40
40
45
20
24
50
30
10
38
35
40
40
24
40
40
40
30

20
30
10

40
40
14
160
28
40
16
16
30
50
40
25
40
40
50
40
55
37
40
40
0
24
40
38
32
36
40
40
15
20
40
37
8
24
34
40
34
38
0
24
20
40

40
32
40
26
16
20
16
40
35
35
30
46
40
35
35
40
40
40
25
40
25
40
40
14
40
24
40
35
40
30
30
40
10
40
10
2
40
25
40
40
28
40

40
30
36
40
40
15
12
40
27
40
40
40
12
36
40
40
40
40
44
40
40
10
40
38
8
43
45
4
40
40
40
0
38
40
4
40
40
40
24
30
40
40

40
40

40
50
40
37
30
16
35
20
40
40
40
40
50
40
40
36
40
50
40
16
40
44
40
28
24

40
29
40
40
30
40
60
40
40
20
40
37
60

32
40
40
20
40
24
40
35
37
40
40
30
38
30
10
24
40
0
35
30
40
8
35
60
15
8
4
35
30
40
35
15
40
0
36
40
30
25
40
40
20
40

20
40
35
40
35
16
24
30
40
40
30
24
35
40
32
40
32
7
40
40
20
30
40
40
40
37
28
40
35
38
25
5
40
25
40
40
30
20
40
40
35
12

32
20
40
45
30
45
40
40
36
30
27
40
40
40
30
40
24
35
40
34
40
40
35
32
37
20
20
40
43
50
77
40
37
37
25
35
8
35
10
40
30
40

24
35
39
40
23
40
40
40
40
40
8
30
30
38
40
44
28
40
34
40
35
8
14
40
45
40
40
40
40
45
20
40
24
8
35
40
18
40
14
40
30
32

40
35
38
35
20
17
45
8
38
40
24
37
40
32
45
40
23
32
38
16
40
40
35
42
15
