# Supplementary material for: Community Pharmacists’ Views and Practices Regarding Natural Health Products Sold in Community Pharmacies
Source: PLoS One. 2016 Sep 23;11(9):e0163450. doi: 10.1371/journal.pone.0163450 (PMC5035072; doi:10.1371/journal.pone.0163450)
Supplement: S3 File — (PDF) [file pone.0163450.s003.pdf]

S3 File. Study results – Cross tabulations

Influence of frequency of NHP recommendations on counselling practices

		In what circumstances do you provide counselling to clients regarding the safety and efficacy of NHPs? Check all that apply.						
		When I recommend a NHP to a client	When a client inquires about a NHP	When a client is picking up a NHP prescribed or recommended by another health care provider	When a client requires assistance locating a NHP in the pharmacy	I have never provided counselling to clients regarding the safety and efficacy of NHPs	Other (please specify):	Total
On a scale of 1 to 5, with 5 being very often and 1 being never, how regularly do you recommend NHPs to clients?	1- Never	36.36%	72.73%	36.36%	18.18%	18.18%	0.00%	100.00%
	2-Rarely	97<br>85.09%	106<br>92.98%	59<br>51.75%	54<br>47.37%	2<br>1.75%	4<br>3.51%	114<br>100.00%
	3-Sometimes	165<br>90.66%	168<br>92.31%	138<br>75.82%	126<br>69.23%	0<br>0.00%	2<br>1.10%	182<br>100.00%
	4-Often	65<br>91.55%	60<br>84.51%	52<br>73.24%	47<br>66.20%	0<br>0.00%	4<br>5.63%	71<br>100.00%
	5-Very Often	21<br>91.30%	20<br>86.96%	19<br>82.61%	17<br>73.91%	0<br>0.00%	1<br>4.35%	23<br>100.00%
	Total	352<br>87.78%	362<br>90.27%	272<br>67.83%	246<br>61.35%	4<br>1.00%	11<br>2.74%	401<br>100.00%

p-value: 0.00

S3 File. Study results – Cross tabulations

NHPs recommended for concurrent use

		What NHPs do you recommend most often? Check all that apply.																							
		Multivitamin/prenatal vitamin	Vitamin B complex	Vitamin B6	Vitamin B12 (oral or sublingual)	Folic acid	Vitamin C	Vitamin D	Calcium	Magnesium	Zinc	Fish oil/omega-3 fatty acids	Melatonin	Iron	Ginseng	Echinacea	Homeopathic products	Probiotics	St. John's wort	Cranberry	Garlic	Gingko biloba	Tea tree oil	Saw palmetto	Psyllium fiber	Weight loss/detoxification products	Other (please specify):
Have you recommended that a client use NHPs concurrently with conventional medicines?	Yes	248<br>73.16%	135<br>39.82%	36<br>10.62%	157<br>46.31%	130<br>38.35%	80<br>23.60%	283<br>83.48%	275<br>81.12%	122<br>35.99%	43<br>12.68%	213<br>62.83%	187<br>55.16%	188<br>55.46%	18<br>5.31%	34<br>10.03%	23<br>6.78%	234<br>69.03%	11<br>3.24%	79<br>23.30%	23<br>6.78%	15<br>4.42%	38<br>11.21%	25<br>7.37%	144<br>42.48%	14<br>4.13%	22<br>6.49%
	No	26<br>63.41%	14<br>34.15%	2<br>4.88%	13<br>31.71%	9<br>21.95%	5<br>12.20%	27<br>65.85%	19<br>46.34%	5<br>12.20%	4<br>9.76%	14<br>34.15%	21<br>51.22%	18<br>43.90%	0<br>0.00%	4<br>9.76%	4<br>9.76%	20<br>48.78%	1<br>2.44%	4<br>9.76%	0<br>0.00%	2<br>4.88%	4<br>9.76%	1<br>2.44%	12<br>29.27%	4<br>9.76%	5<br>12.20%
	Not Sure	12<br>70.59%	4<br>23.53%	0<br>0.00%	7<br>41.18%	6<br>35.29%	5<br>29.41%	15<br>88.24%	14<br>82.35%	5<br>29.41%	2<br>11.76%	9<br>52.94%	4<br>23.53%	13<br>76.47%	3<br>17.65%	0<br>0.00%	1<br>5.88%	10<br>58.82%	0<br>0.00%	4<br>23.53%	2<br>11.76%	2<br>11.76%	1<br>5.88%	0<br>0.00%	3<br>17.65%	0<br>0.00%	0<br>0.00%
	Total	286<br>72.04%	153<br>38.54%	38<br>9.57%	177<br>44.58%	145<br>36.52%	90<br>22.67%	325<br>81.86%	308<br>77.58%	132<br>33.25%	49<br>12.34%	236<br>59.45%	212<br>53.40%	219<br>55.16%	21<br>5.29%	38<br>9.57%	28<br>7.05%	264<br>66.50%	12<br>3.02%	87<br>21.91%	25<br>6.30%	19<br>4.79%	43<br>10.83%	26<br>6.55%	159<br>40.05%	18<br>4.53%	27<br>6.80%

p-value: 0.03

S3 File. Study results – Cross tabulations

NHP recommendations by practice setting

		What NHPs do you recommend most often? Check all that apply.																										
		Multivitamin/prenatal vitamin	Vitamin B complex	Vitamin B6	Vitamin B12 (oral or sublingual)	Folic acid	Vitamin C	Vitamin D	Calcium	Magnesium	Zinc	Fish oil/omega-3 fatty acids	Melatonin	Iron	Ginseng	Echinacea	Homeopathic products	Probiotics	St. John's wort	Cranberry	Garlic	Gingko biloba	Tea tree oil	Saw palmetto	Psyllium fiber	Weight loss/detoxification products	Other (please specify):	Total
Please indicate your practice setting.	Rural	69<br>68.32%	38<br>37.62%	7<br>6.93%	50<br>49.50%	39<br>38.61%	27<br>26.73%	84<br>83.17%	82<br>81.19%	48<br>47.52%	21<br>20.79%	56<br>55.45%	58<br>57.43%	54<br>53.47%	8<br>7.92%	13<br>12.87%	8<br>7.92%	78<br>77.23%	4<br>3.96%	23<br>22.77%	13<br>12.87%	5<br>4.95%	13<br>12.87%	11<br>10.89%	46<br>45.54%	6<br>5.94%	9<br>8.91%	101<br>100.00%
	Urban	218<br>73.15%	116<br>38.93%	31<br>10.40%	128<br>42.95%	106<br>35.57%	64<br>21.48%	242<br>81.21%	228<br>76.51%	86<br>28.86%	28<br>9.40%	181<br>60.74%	154<br>51.68%	167<br>56.04%	13<br>4.36%	26<br>8.72%	20<br>6.71%	187<br>62.75%	8<br>2.68%	66<br>22.15%	13<br>4.36%	15<br>5.03%	30<br>10.07%	15<br>5.03%	114<br>38.26%	12<br>4.03%	18<br>6.04%	298<br>100.00%
	Total	287<br>71.93%	154<br>38.60%	38<br>9.52%	178<br>44.61%	145<br>36.34%	91<br>22.81%	326<br>81.70%	310<br>77.69%	134<br>33.58%	49<br>12.28%	237<br>59.40%	212<br>53.13%	221<br>55.39%	21<br>5.26%	39<br>9.77%	28<br>7.02%	265<br>66.42%	12<br>3.01%	89<br>22.31%	26<br>6.52%	20<br>5.01%	43<br>10.78%	26<br>6.52%	160<br>40.10%	18<br>4.51%	27<br>6.77%	399<br>100.00%

p-value: 0.02

S3 File. Study results – Cross tabulations

NHP recommendations by source of recommendation

		What NHPs do you recommend most often? Check all that apply.																									
		Multivitamin/prenatal vitamin	Vitamin B complex	Vitamin B6	Vitamin B12 (oral or sublingual)	Folic acid	Vitamin C	Vitamin D	Calcium	Magnesium	Zinc	Fish oil/omega-3 fatty acids	Melatonin	Iron	Ginseng	Echinacea	Homeopathic products	Probiotics	St. John's wort	Cranberry	Garlic	Gingko biloba	Tea tree oil	Saw palmetto	Psyllium fiber	Weight loss/detoxification products	Other (please specify):	Total
What is your primary basis for recommending NHPs to clients? Check one.	Health Canada approval of NHP	39<br>81.25%	23<br>47.92%	9<br>18.75%	23<br>47.92%	20<br>41.67%	16<br>33.33%	39<br>81.25%	38<br>79.17%	19<br>39.58%	8<br>16.67%	28<br>58.33%	26<br>54.17%	29<br>60.42%	4<br>8.33%	7<br>14.58%	6<br>12.50%	32<br>66.67%	1<br>2.08%	15<br>31.25%	3<br>6.25%	4<br>8.33%	3<br>6.25%	3<br>6.25%	13<br>27.08%	4<br>8.33%	0<br>0.00%	48<br>100.00%
	Manufacturer information	12<br>100.00%	10<br>83.33%	2<br>16.67%	3<br>25.00%	5<br>41.67%	3<br>25.00%	6<br>50.00%	6<br>50.00%	6<br>50.00%	3<br>25.00%	8<br>66.67%	7<br>58.33%	5<br>41.67%	3<br>25.00%	3<br>25.00%	4<br>33.33%	10<br>83.33%	2<br>16.67%	4<br>33.33%	4<br>33.33%	2<br>16.67%	3<br>25.00%	3<br>25.00%	4<br>33.33%	2<br>16.67%	2<br>16.67%	12<br>100.00%
	Primary literature	32<br>58.18%	18<br>32.73%	4<br>7.27%	19<br>34.55%	18<br>32.73%	10<br>18.18%	43<br>78.18%	33<br>60.00%	15<br>27.27%	11<br>20.00%	27<br>49.09%	26<br>47.27%	28<br>50.91%	1<br>1.82%	4<br>7.27%	3<br>5.45%	34<br>61.82%	0<br>0.00%	12<br>21.82%	3<br>5.45%	1<br>1.82%	3<br>5.45%	3<br>5.45%	15<br>27.27%	2<br>3.64%	8<br>14.55%	55<br>100.00%
	Review articles (i.e. Pharmacist's Letter, CPJ)	102<br>72.86%	49<br>35.00%	11<br>7.86%	67<br>47.86%	52<br>37.14%	28<br>20.00%	123<br>87.86%	119<br>85.00%	44<br>31.43%	15<br>10.71%	94<br>67.14%	74<br>52.86%	77<br>55.00%	7<br>5.00%	12<br>8.57%	6<br>4.29%	97<br>69.29%	6<br>4.29%	26<br>18.57%	9<br>6.43%	8<br>5.71%	12<br>8.57%	6<br>4.29%	74<br>52.86%	3<br>2.14%	7<br>5.00%	140<br>100.00%
	Client request	71<br>76.34%	42<br>45.16%	9<br>9.68%	36<br>38.71%	30<br>32.26%	22<br>23.66%	70<br>75.27%	77<br>82.80%	27<br>29.03%	6<br>6.45%	49<br>52.69%	54<br>58.06%	51<br>54.84%	4<br>4.30%	9<br>9.68%	4<br>4.30%	57<br>61.29%	0<br>0.00%	21<br>22.58%	1<br>1.08%	3<br>3.23%	16<br>17.20%	5<br>5.38%	39<br>41.94%	2<br>2.15%	5<br>5.38%	93<br>100.00%
	Recommendation/prescription from primary care provider	21<br>67.74%	8<br>25.81%	1<br>3.23%	18<br>58.06%	14<br>45.16%	6<br>19.35%	30<br>96.77%	26<br>83.87%	12<br>38.71%	4<br>12.90%	17<br>54.84%	16<br>51.61%	20<br>64.52%	2<br>6.45%	3<br>9.68%	4<br>12.90%	23<br>74.19%	3<br>9.68%	8<br>25.81%	4<br>12.90%	2<br>6.45%	2<br>6.45%	3<br>9.68%	11<br>35.48%	2<br>6.45%	1<br>3.23%	31<br>100.00%
	Internet/social media	1<br>50.00%	0<br>0.00%	0<br>0.00%	1<br>50.00%	1<br>50.00%	1<br>50.00%	2<br>100.00%	0<br>0.00%	0<br>0.00%	0<br>0.00%	1<br>50.00%	0<br>0.00%	0<br>0.00%	0<br>0.00%	0<br>0.00%	0<br>0.00%	1<br>50.00%	0<br>0.00%	0<br>0.00%	0<br>0.00%	0<br>0.00%	0<br>0.00%	0<br>0.00%	0<br>0.00%	0<br>0.00%	0<br>0.00%	2<br>100.00%
	Other (please specify):	9<br>50.00%	4<br>22.22%	2<br>11.11%	11<br>61.11%	5<br>27.78%	5<br>27.78%	13<br>72.22%	11<br>61.11%	11<br>61.11%	2<br>11.11%	13<br>72.22%	9<br>50.00%	11<br>61.11%	0<br>0.00%	1<br>5.56%	1<br>5.56%	11<br>61.11%	0<br>0.00%	3<br>16.67%	2<br>11.11%	0<br>0.00%	4<br>22.22%	3<br>16.67%	4<br>22.22%	3<br>16.67%	4<br>22.22%	18<br>100.00%
	Total	287<br>71.93%	154<br>38.60%	38<br>9.52%	178<br>44.61%	145<br>36.34%	91<br>22.81%	326<br>81.70%	310<br>77.69%	134<br>33.58%	49<br>12.28%	237<br>59.40%	212<br>53.13%	221<br>55.39%	21<br>5.26%	39<br>9.77%	28<br>7.02%	265<br>66.42%	12<br>3.01%	89<br>22.31%	26<br>6.52%	20<br>5.01%	43<br>10.78%	26<br>6.52%	160<br>40.10%	18<br>4.51%	27<br>6.77%	399<br>100.00%

p-value: 0.00

S3 File. Study results – Cross tabulations

NHP recommendations by NHP learning

		What NHPs do you recommend most often? Check all that apply.																										
		Multivitamin/prenatal vitamin	Vitamin B complex	Vitamin B6	Vitamin B12 (oral or sublingual)	Folic acid	Vitamin C	Vitamin D	Calcium	Magnesium	Zinc	Fish oil/omega-3 fatty acids	Melatonin	Iron	Ginseng	Echinacea	Homeopathic products	Probiotics	St. John's wort	Cranberry	Garlic	Gingko biloba	Tea tree oil	Saw palmetto	Psyllium fiber	Weight loss/detoxification products	Other (please specify):	Total
Please indicate the number of hours you have spent on accredited and non-accredited NHP learning within the past two years	None	40<br>70.18%	19<br>33.33%	2<br>3.51%	21<br>36.84%	12<br>21.05%	12<br>21.05%	40<br>70.18%	38<br>66.67%	12<br>21.05%	5<br>8.77%	26<br>45.61%	27<br>47.37%	31<br>54.39%	1<br>1.75%	4<br>7.02%	5<br>8.77%	36<br>63.16%	2<br>3.51%	12<br>21.05%	1<br>1.75%	1<br>1.75%	4<br>7.02%	2<br>3.51%	13<br>22.81%	2<br>3.51%	6<br>10.53%	57<br>100.00%
	1 to 3 hours	97<br>71.85%	50<br>37.04%	16<br>11.85%	63<br>46.67%	54<br>40.00%	24<br>17.78%	112<br>82.96%	106<br>78.52%	40<br>29.63%	12<br>8.89%	75<br>55.56%	70<br>51.85%	81<br>60.00%	8<br>5.93%	13<br>9.63%	4<br>2.96%	86<br>63.70%	3<br>2.22%	26<br>19.26%	9<br>6.67%	8<br>5.93%	10<br>7.41%	8<br>5.93%	59<br>43.70%	6<br>4.44%	5<br>3.70%	135<br>100.00%
	4 to 6 hours	55<br>71.43%	28<br>36.36%	7<br>9.09%	39<br>50.65%	30<br>38.96%	17<br>22.08%	62<br>80.52%	63<br>81.82%	20<br>25.97%	4<br>5.19%	50<br>64.94%	41<br>53.25%	42<br>54.55%	2<br>2.60%	4<br>5.19%	4<br>5.19%	48<br>62.34%	0<br>0.00%	15<br>19.48%	3<br>3.90%	3<br>3.90%	8<br>10.39%	3<br>3.90%	30<br>38.96%	3<br>3.90%	5<br>6.49%	77<br>100.00%
	7 to 10 hours	15<br>65.22%	10<br>43.48%	5<br>21.74%	11<br>47.83%	9<br>39.13%	10<br>43.48%	20<br>86.96%	20<br>86.96%	11<br>47.83%	7<br>30.43%	15<br>65.22%	17<br>73.91%	15<br>65.22%	0<br>0.00%	3<br>13.04%	4<br>17.39%	14<br>60.87%	3<br>13.04%	9<br>39.13%	3<br>13.04%	0<br>0.00%	8<br>34.78%	4<br>17.39%	10<br>43.48%	1<br>4.35%	2<br>8.70%	23<br>100.00%
	More than 10 hours	80<br>75.47%	46<br>43.40%	8<br>7.55%	44<br>41.51%	40<br>37.74%	28<br>26.42%	91<br>85.85%	83<br>78.30%	51<br>48.11%	21<br>19.81%	71<br>66.98%	56<br>52.83%	52<br>49.06%	10<br>9.43%	15<br>14.15%	11<br>10.38%	80<br>75.47%	4<br>3.77%	27<br>25.47%	10<br>9.43%	8<br>7.55%	13<br>12.26%	9<br>8.49%	48<br>45.28%	6<br>5.66%	9<br>8.49%	106<br>100.00%
	Total	287<br>72.11%	153<br>38.44%	38<br>9.55%	178<br>44.72%	145<br>36.43%	91<br>22.86%	325<br>81.66%	310<br>77.89%	134<br>33.67%	49<br>12.31%	237<br>59.55%	211<br>53.02%	221<br>55.53%	21<br>5.28%	39<br>9.80%	28<br>7.04%	264<br>66.33%	12<br>3.02%	89<br>22.36%	26<br>6.53%	20<br>5.03%	43<br>10.80%	26<br>6.53%	160<br>40.20%	18<br>4.52%	27<br>6.78%	398<br>100.00%

p-value: 0.01
